# High flow nasal cannula oxygen (hfnc) versus non-invasive positive pressure ventilation (nippv) in acute hypoxemic respiratory failure. a pilot randomized controlled trial

**DOI:** 10.1186/2197-425X-3-S1-A166

**Published:** 2015-10-01

**Authors:** JR Azevedo, WS Montenegro, AL Leitao, MM Silva, JS Prazeres, JP Maranhao

**Affiliations:** Hospital Sao Domingos, Intensive Care Unit, Sao Luis, Brazil

## Introduction

Oxygen therapy is generally provided via nasal cannula, non-rebreathing masks and masks with reservoir bags. These devices have various limitations.

Despite showing clear benefits in certain conditions, non-invasive positive pressure ventilation (NIPPV) limits mobilization, restricts communication and oral nutrition and is poorly tolerated by some patients due to discomfort.

High-flow nasal cannula oxygen (HFNC) delivers a high flow of heated and humidified oxygen through nasal prongs. It generate positive airway pressure, reduces respiratory dead space, airway resistance, and less frequent interruption of therapy.

## Objectives

The primary aim of this study was to compare HFNC and NIPPV efficacy to prevent endotracheal intubation in patients with acute hypoxemic respiratory failure. Secondarily to access comfort, ventilatory and oxygenation parameters.

## Methods

We included all adult patients admitted to a 45-bed medical surgical ICU in the period from December 2013 to March 2015, with acute hypoxemic respiratory failure defined as SpO2 < 95% while receiving oxygen through a facemask at an estimated FIO2= 50%. The study was approved by the Ethics Committee of Sao Domingos Hospital.

HFNC oxygen was delivered via a high flow delivery system (Optiflow; Fisher &Paykel, Aukland New Zeland) and an air-oxygen blender that delivers a gas flow of up to 60 liters/min to a heated humidifier (Fisher&Paykel MR 850).

NIPPV was delivered with a full-face mask (Performax, Philips Respironics), connected to an ICU ventilator with a NIV mode Patients were ventilated in the mode pressure support.

Comfort was evaluated through modified Borg scale at baseline and at the end of therapy. Respiratory rate and SpO2/FIO2 were measured at baseline and after 6, 12 and 24 hours of support.

Clinical stabilization was defined as a SpO2 > 94% and respiratory rate < 28 rpm with a low flow oxygen nasal catheter or Venturi ≤ 0.40. Successful treatment was defined by avoidance of intubation.

## Results

**Figure 1:**
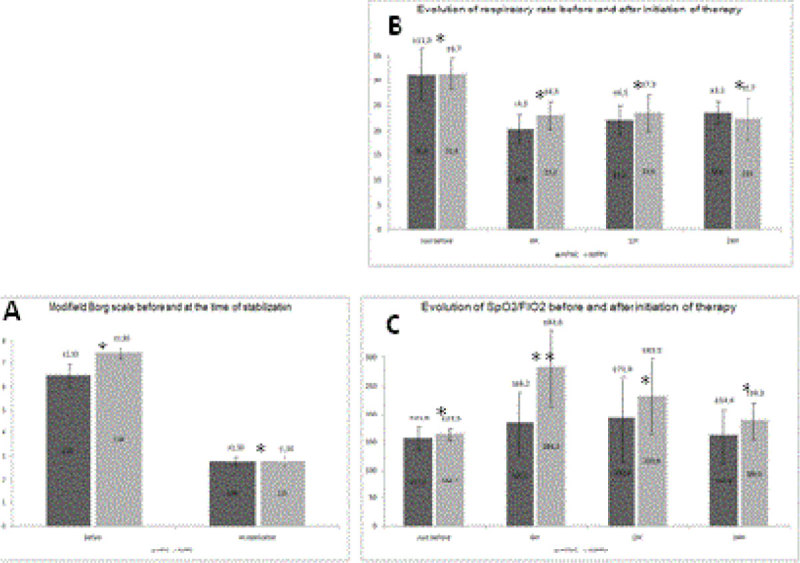
Figure 1

Thirty-five patients were submitted to randomization. Five were excluded from analysis. Thus, 30 patients were analyzed, 14 in HFNC group and 16 in NIPPV group. Both groups were comparable regarding to age, gender and APACHE IV score (Table 1). Twelve patients were intubated due to failure of therapy (6 in each group). Three interruptions of therapy were motivated by patient intolerance (2 NIPPV and 1 HFNC). Modified Borg scale improved substantially in both groups (Figure 1 Panel A). Improvement of respiratory rate and PbO2/FIO2 also were expressive in both groups after beginning therapy (Figure 1 Panel B and C).

**Table 1:** Characteristcs of study population.

	HFNC (N = 14)	NIPPV (N = 16)	P			HFNC (N = 14)	NIPPV (N = 16)	P	
Female, n (%)	8 (57)	8 (50)			Duration of therapy, hours median Interquartile range	24.00 14.04-59.04	17.00 8.86-35.14	0.38	
Age, y, mean (SD)	61,4 (13.7)	72.3 (19.0)	0.08		Outcome Intubation	9 (64.2)	9 (56.2)	0.72	
APACHE IV, mean (SD)	58.3 (17.2)	65.5 (23.5)	0.33						
Etiology of ARF									
Pulmonary edema (cardiogenic)	4	9							
Community-acquiered pneumonia	5	5							
Sepsis	2	-							
Aspiration	2	-							
Other	1	2							

## Conclusions

We found no significant difference in the necessity of endotracheal intubation and invasive ventilation in patients with hypoxemic respiratory failure managed with HFNC compared to NIPPV.

